# Thunderclap Headache in a Patient With Giant Cell Arteritis: Consider Aortic Dissection

**DOI:** 10.1002/ccr3.73178

**Published:** 2026-07-22

**Authors:** Alice Lee, Joanna Myszkowska, Sian Finlay, Eleanor Gasse, Chris Isles

**Affiliations:** ^1^ Acute Medical Unit Dumfries and Galloway Royal Infirmary Dumfries UK; ^2^ Next of Kin Vale Guernsey

**Keywords:** aortic dissection, Aortitis, Giant cell arteritis, thunderclap headache, worst ever headache

## Abstract

Thunderclap is a rare but important manifestation of type A aortic dissection and may occur as the sole presenting feature in patients who do not experience chest pain.

## 1 Introduction

Thunderclap headache is usually described as a “worst ever” headache of sudden onset that reaches maximal intensity within 1 min [1, 2, 3, 4, 5]. The classic cause of thunderclap headache is subarachnoid hemorrhage, which accounts for around one in 10 of sudden onset severe headache seen in the emergency department [6]. Important other causes of thunderclap headache highlighted in a recent review include intracranial hemorrhage, acute ischemic stroke, intracranial or extracranial arterial dissection, cerebral venous sinus thrombosis, pituitary apoplexy, spontaneous intracranial hypotension due to CSF leak, reversible cerebral vasoconstriction syndrome, meningitis, and acute angle closure glaucoma [7].

Conspicuous by their absence from this and other reviews [8, 9, 10, 11, 12] are the thunderclap headaches that can occur with giant cell arteritis (GCA) [13, 14, 15] and aortic dissection [16, 17, 18, 19, 20, 21, 22]. We found 7 case reports of patients whose thunderclap headache was a consequence of aortic dissection and who did not experience chest pain at the onset of or immediately preceding their headache [16, 17, 18, 19, 20, 21, 22] (Table 1). The omission of a link between aortic dissection and thunderclap headache has since been noted in a response to the Rosenberg review [23]. Against this background, we wish to report a case of a patient presenting with thunderclap headache due to type A aortic dissection, which we did not suspect during life and which was only confirmed at autopsy. The patient never complained of chest pain.

**TABLE 1:** Case reports of patients who did not report chest pain at the onset of headache.

	Age/sex	Headache	Chest pain at presentation	Additional clues	Type of dissection
Stollberger 1998	61 F	Continuous, bifrontal	No	Right‐sided CP, new diastolic murmur two hours later	Type A
Mathys 2004	53 M	Severe frontal with neck pain	No	Anterior CP for 10 min the day before, new diastolic murmur	Type A
Nohe 2005	63 F	Sudden onset, excruciating	No	D‐dimer 9000ug/l, CXR broad mediastinum	Type A
Singh 2006	50 M	Sudden onset, severe, bifrontal	No	Borderline widened mediastinum, BP difference arms > 10 mmHg	Type A
Rust 2013	67 F	Thunderclap	No	None	Type A
Chahine 2018	44 M	Rapidly progressive, severe, frontal	No	Transient CP and left leg numbness 48 h later, new diastolic murmur	Type A
Ranjeevan 2025	54 F	Thunderclap, starting neck, radiating to top of head	No	Transient sensory and motor limb deficits, slight abdominal discomfort	Type A

## 2 Case History and Examination

A 76‐year‐old man presented in September 2024 with thunderclap headache, onset while bending, maximal intensity within 1 min, and severity 8/10 initially. Past history included GCA first diagnosed in June 2022 for which he had completed a course of steroid 8 months previously. Aortic involvement was neither suspected nor screened during or after steroid treatment. He was also known to be hypertensive and was taking lisinopril 10 mg once daily for this. He felt slightly clammy when first seen but had no other symptoms, and in particular, he did not experience chest or back pain, and nor was he breathless. Blood pressure was 170/90 mmHg initially, settling to 146/66 mmHg. We did not check for a pulse deficit. There were no murmurs and no focal neurology.

## 3 Investigations and Treatment

CT brain undertaken within 6 h of the onset of headache showed no evidence of intracranial hemorrhage, large territory infarction, or mass lesion. The results of other investigations included Hb 119 g/L, serum potassium 4.4 mmol/L, blood urea 5.7 mmol/L, and serum creatinine 88 umol/L. CRP was 9 mg/L (NR 0–10) while ESR was 13 mm/h (NR 0–20). ECG showed sinus rhythm 50/min. We did not request CXR or D‐dimer.

## 4 Outcome

The patient's headache improved to 1/10 severity with paracetamol, which led us to discharge him with worsening advice after 3 h observation, only for him to die suddenly at home a few hours later. Autopsy revealed that the cause of his presentation was a type A aortic dissection with an intimal tear 50 mm from the aortic valve and a false lumen that extended 90 mm toward the aortic arch. External rupture of the aortic wall was observed with significant hemorrhage into surrounding soft tissue and around the structures of the mediastinum and lower neck. There was no evidence of carotid artery dissection, intracerebral, or subarachnoid hemorrhage. The pathologist felt that the total volume of extravasated blood was significant and that the likely cause of death was hemorrhagic shock.

## 5 Discussion

The relation between GCA, aortic dissection, and thunderclap headache is interesting, not only because GCA [13, 14, 15] and aortic dissection [16, 17, 18, 19, 20, 21, 22] can cause thunderclap headache but also because aortic dissection is more common in patients with GCA [24, 25]. Headache as the initial manifestation of aortic dissection may be a consequence of vessel wall distension, ischaemia of the pericarotid plexus, or decreased cerebral perfusion [22].

We did not consider the possibility of aortic dissection in our patient, but are unlikely to be alone in failing to do so, as neither GCA nor aortic dissection were recognized as causes of thunderclap headache in recent reviews [7, 8, 9, 10, 11, 12]. Sjulstad et al. in a study of 588 patients presenting to their ED with thunderclap headache noted that most were discharged with a diagnosis of nonspecific headache after first ruling out SAH [26]. Our case had no clinical features that might have prompted vascular imaging, although his past history of GCA should, with hindsight, have led us to consider further assessment. Moreover, we should not have been deterred by the fact that his GCA appeared inactive as it is well recognized that involvement of the aorta and its proximal branches may be asymptomatic [9] and that aortitis can persist for years even when a patient appears to be in full remission [27].

Notwithstanding, we do not believe it would be cost‐effective to request CT aorta for all patients with thunderclap headache in whom SAH has been excluded. The yield would be extremely low. A better approach might be to combine the Aortic Dissection Detection Risk Score (ADD‐RS) with D‐dimer and consider further assessment of thunderclap headache based on the patient's underlying conditions (e.g., GCA, Marfan's syndrome), the nature of their pain (e.g., tearing chest pain that radiates to the back), and examination findings (e.g., new diastolic murmur or a BP difference greater than 10 mmHg between the arms). Low‐risk patients who have fewer than two of these features could have their D‐dimer measured, while those with two or more features might go straight to aortography [28]. D‐dimer is usually elevated in AD, while a normal D‐dimer in a low‐risk patient effectively rules AD out [28, 29].

In summary, we believe our case, together with those previously reported, supports the view that aortic dissection should be added to the differential diagnosis of thunderclap headache, particularly if the patient also happens to have a history of GCA.

## Author Contributions

**Alice Lee:** writing – original draft, writing – review and editing. **Sian Finlay:** writing – review and editing. **Joanna Myszkowska:** investigation, writing – review and editing. **Eleanor Gasse:** validation. **Chris Isles:** conceptualization, supervision, writing – original draft, writing – review and editing.

## Funding

The authors have nothing to report.

## Ethics Statement

We did not seek ethics approval as no patient identifiable data were included, in keeping with our health board's policy.

## Consent

Written informed consent was obtained from the patient's next of kin, according to journal guidelines.

## Conflicts of Interest

The authors declare no conflicts of interest.

## Data Availability

Further details of our patient's history can be made available if required and upon request.
